# Systemizers Are Better Code-Breakers: Self-Reported Systemizing Predicts Code-Breaking Performance in Expert Hackers and Naïve Participants

**DOI:** 10.3389/fnhum.2016.00229

**Published:** 2016-05-17

**Authors:** India Harvey, Samuela Bolgan, Daniel Mosca, Colin McLean, Elena Rusconi

**Affiliations:** ^1^Division of Psychology, Abertay UniversityDundee, UK; ^2^Division of Arts Media and Computer Games, Abertay UniversityDundee, UK; ^3^Department of Security and Crime Sciences, University College LondonLondon, UK

**Keywords:** systemizing, attention to detail, autism spectrum disorders, hacking, code breaking, security

## Abstract

Studies on *hacking* have typically focused on motivational aspects and general personality traits of the individuals who engage in hacking; little systematic research has been conducted on predispositions that may be associated not only with the choice to pursue a hacking career but also with performance in either naïve or expert populations. Here, we test the hypotheses that two traits that are typically enhanced in autism spectrum disorders—attention to detail and systemizing—may be positively related to both the choice of pursuing a career in information security and skilled performance in a prototypical hacking task (i.e., crypto-analysis or *code-breaking*). A group of naïve participants and of ethical hackers completed the Autism Spectrum Quotient, including an attention to detail scale, and the Systemizing Quotient (Baron-Cohen et al., 2001, 2003). They were also tested with behavioral tasks involving code-breaking and a control task involving security X-ray image interpretation. Hackers reported significantly higher systemizing and attention to detail than non-hackers. We found a positive relation between self-reported systemizing (but not attention to detail) and code-breaking skills in both hackers and non-hackers, whereas attention to detail (but not systemizing) was related with performance in the X-ray screening task in both groups, as previously reported with naïve participants (Rusconi et al., 2015). We discuss the theoretical and translational implications of our findings.

## Introduction

### The information security challenge

It is estimated that there were 3,035,749,340 internet users in a global population of 7,182,406,565 individuals (i.e., 42.3%) as of mid-June 2014. The overall usage of the world-wide-web has increased of 753% over the last 15 years (Internetworldstats.Com). With the mass diffusion of computers and internet access, digital information security (e.g., ensuring the confidentiality and authenticity of digital messages and preventing unauthorized access to information stored in digital format) has become a major topic of concern for businesses, governments, and individuals. A common solution to this challenge is offered by cryptography which, in its most current form, makes use of complex mathematical algorithms to prevent unauthorized access to or tampering with digital information (Menzes et al., 1996; Singh, 1999; Piper and Murphy, 2002). The process of transforming a piece of information in such a way that it results unintelligible to unauthorized users is called encryption. If encrypted information was intercepted by someone other than the intended user or recipient (which is especially likely when it travels on the internet), it should still remain unintelligible to users who do not possess an appropriate decryption key. Encryption is a very powerful tool but keys and algorithms may have a limited lifespan due to advancements in the technology available to both cryptanalysts and interceptors (i.e., individuals who attempt to deduce the content of encrypted information without possessing a decryption key)—thus the standard encryption toolset is vulnerable and requires continuous updating.

Every year, a multitude of companies and individuals are victims of malicious hackers who succeed in penetrating networks and accessing sensitive data. According to a study published by McAfee's Centre for Strategic and International Studies (McAfee, 2014) hackers are costing consumers and companies between $375 and $575 billion per year. One of the biggest hurdles in digital information security is the ever evolving character of threats. Most of these threats are developed by skilled individuals with access to the internet and a good background knowledge of computer science and network security issues. These skills enable them to obtain restricted-access information and tamper and exploit it to their aims if they wish so. The cyber security industry offers a series of tools to counter the threat and minimize the residual risk. These range from secure encryption methods, firewalls, automated intrusion detection, and prevention systems. However, because cybercriminals are thinking adversaries, many measures to improve security often beget countermeasures.

Arguably, an effective way to evaluate and counter intruder threats to organizations would be to have independent computer security professionals attempt to break into computer systems (i.e., penetration testers; Palmer, 2001). Both individuals who develop and use cyber-threats against systems and individuals who perform authorized penetration testing engage in *hacking*. As malicious hacking grows, businesses, and governments have started to hire hackers and penetration testers to help probe and improve their networks, applications, and computer systems with the ultimate goal of preventing data theft and fraud. Modern day terminology often titles them as ethical hackers, IT security analysts, information security professionals, cybersecurity/computer security specialists, or information security analysts.

### Individual predispositions to hacking: the potential of autistic traits

At present, the literature lacks of empirical research on the hacker mind-set, especially studies involving behavioral evidence on abilities and predispositions (Zhengchuan et al., 2013). More evidence is available on the cognitive skills that are needed to achieve proficiency in computer programming, which is normally part of a hacker's set of skills. Across studies, spatial ability and spatial reasoning, often measured with mental rotation tests, were shown to be positively correlated with computer programming ability (e.g., Wilson and Shrock, 2001; Jones and Burnett, 2008; Ambrosio et al., 2014). Performance in visuospatial perception tasks, such as pattern recognition, and in particular the ability to find similarities between dissimilar items and to detect internal order in a series, is predictive of programming performance (e.g., Subramanian and Joshi, 1996). Field independency, as measured using variants of the embedded figures test, appears to be a critical skill in learning to program (Mancy and Reid, 2004). It is also widely accepted that programming requires using mental models of the computing environment (Canas et al., 1994; Mayer, 1981; Young, 1981). A mental model refers to the user's mental representation of the components and operating rules of a system; it may vary with respect to its completeness and correspondence with reality (Mayer, 1981). A good mental model requires deep understanding of how different components (e.g., input system, memory, program, output system, etc.) interact to produce specific actions (Canas et al., 1994) and several studies have shown that mental model training increases subjects' programming performance (Mayer, 1981).

However, the approach of hackers to computer programming may partly differ from the approach of programmers. According to Bratus (2007), programmers learn to interpret and fix errors as well as to avoid situations that cause opportunities to introduce errors. They usually remain in the dark about the actual mechanisms that cause the errors; hackers are fully aware of such causes and develop tools for examining and manipulating them. This difference is ascribed to differences in their curriculum and training: developers and programmers are typically confined to work within narrow models of computing environment for better productivity and compatibility with existing structures. On the other hand, hackers are trained to go beyond those boundaries, develop a lateral way of thinking, and a detailed/comprehensive mental model of the system enabling them to detect any vulnerability. In other words hackers, whose programming skills are functional to digital information gathering, penetration testing and code-breaking, are prone to reason and to test new hypotheses at the system level. For any of these tasks, they are trained to identify regularities/patterns in a system's concept and implementation, grasp their implications in terms of possible exploits, probe entry points, and overtake control.

All of these characteristics suggest potentially strong connections between hacking skills and the positive traits of autism. Indeed, people with autism-spectrum disorders tend to show superior abilities in tasks requiring field-independence, analysing spatial relations, detecting patterns, working with systems (Baron-Cohen, 2002); they also appear to be especially drawn to occupations and cultivate interests in STEM disciplines (Baron-Cohen et al., 1999, 2001). Anecdotal evidence shows that people with Asperger's syndrome may perform well and thrive in IT and computer programming jobs (Putzier, 2013). Research has shown that they are capable of teaching themselves and developing hacking skills without receiving any professional training (Kushner, 2011).

Their spontaneous attention to detail could predispose them to hacking by triggering a drive to build and understand systems (i.e., *systemizing*). Baron-Cohen et al. (2003) defined systemizing as the drive to analyse, understand, predict, control, and construct rule-based systems. Systems could belong to different categories: technical, natural, abstract, social, organizable, and/or motoric. However, they all share the same underlying processes in a tripartite structure: input 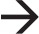 operation 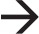 output (Baron-Cohen et al., 2003; Billington et al., 2007). Dealing with systems means examining relationships between components and correlations between events in order to detect any underlying regularities and identify rules (Lawson et al., 2004). Baron-Cohen et al. (2009) maintain that piecemeal attention is instrumental and necessary to the development of skills in systemizing domains but it is not sufficient by itself; without the drive to identify patterns, test, and apply rules the individual would remain lost in the details rather than build coherent and predictive mental models. An alternative proposal maintains that the autistic tendency to cultivate narrow interests and engage in repetitive behaviors would allow certain individuals to achieve excellence in particular fields by extensive practice (e.g., Ericsson and Faivre, 1988). Finally, skill development in people with autism may also be fostered by the co-occurrence of mind-blindness for others' and/or for their own mind (which causes typical social and communication impairments) and their detail-focused cognition (see Happé and Vital, 2009 for an insightful discussion of this point). In summary, from the autism literature we derive the hypothesis that heightened attention to detail may not suffice to predispose an individual to hacking, whereas systemizing may possibly play a more crucial role. In addition, repetitive interests (Ericsson and Faivre, 1988) or social-communication impairments (Happé and Vital, 2009) could also contribute.

According to the *continuum* view of autism, the individual expression of autistic traits varies on a continuum from normality to full-blown autism, which is characterized by the concomitant presence of piecemeal attention, defective social and communication development, repetitive behavior and restricted imagination (DSM-5; Wing, 1981; Frith, 1991; Baron-Cohen, 1995). It follows that in the general population, certain individuals may express a detail-focused cognitive profile, and some of them would develop a drive to systemize more strongly than others (Baron-Cohen et al., 2009). In line with this reasoning, we would expect these individuals to be particularly suited to the hacking job. Moreover, because the traits that co-occur in autism-spectrum disorders may be the expression of independent sets of genes (Happé et al., 2006), neurotypical individuals offer the opportunity to study detail-focus in dissociation from social/communication impairments, narrow interests, and repetitive behaviors (see also Rusconi et al., 2012, 2015). A practical way to measure autistic traits in the general population is via self-reports, for example by asking individuals to rate how well a series of statements tapping domains in which people with autism deviate from the neurotypical population applies to their own cognitive and behavioral profile. One such tool is the Autism Spectrum Quotient (*AQ*; Baron-Cohen et al., 2001). The *AQ* contains 50 statements tapping five different domains, whose scores may indicate autism-like cognition: attention to detail, attention switching, imagination, communication, and social skills. Every domain is assessed by 10 items, and higher scores are obtained by individuals endorsing the autistic-like option. Higher scores in every domain will thus characterize individuals with hyper-attention to detail and poor attention switching, imagination, communication, and social skills.

Schell and Melnychuk (2011) reported testing a sample of hacker conference attendees with the *AQ*. They found that the majority of their sample obtained *AQ* scores falling in the upper half of the distribution of scores for the general population as reported by Baron-Cohen et al. (2001; see also Ruzich et al., 2015) but still much lower than the scores obtained by people with autism. Interestingly, of the five domains contributing to the total *AQ* the one that the hacker conference attendees agreed with the most indicated exceptional attention to local details. In addition, five of the six items that the overall group of hacker conference attendees agreed with the most belong to the attention to detail subscale. This supports the hypothesis of a connection between a detail-focused cognitive profile and interest in hacking. To our knowledge, no study has previously attempted to test (1) the relation between self-reported autistic traits (including systemizing) and prototypical hacking skills (e.g., code-breaking) and (2) identify which autism-related traits may predict hacking performance.

### This study

In this study we addressed three main general questions: (a) are autistic traits higher in ethical hacking students compared to non-hackers; (b) are they related with actual hacking skills in both hackers and non-hackers, and if so how specific is their relation; (c) is detail-focus sufficient to predict hacking skills in hackers and non-hackers. In the current section we present our set of measures and hypotheses. Traits were measured via self-reports and skills via behavioral tasks. Our study tests both correlations amongst several individual traits and also between individual traits and behavioral performance. The study does not test causal connections.

We collected *AQ* scores from two groups of participants: hackers and non-hackers. We predicted that hackers would be characterized by higher detail-focus (Baron-Cohen et al., 2009; Schell and Melnychuk, 2011) and thus predicted that their Attention to Detail scores would be significantly higher than non-hackers' scores; we also tested whether hackers and non-hackers differed in their scores for other scales (Attention Switching, Communication, Imagination, and Social Skills) to assess whether other typical autistic traits such as restricted interests and social/communication weaknesses may characterize the hacker mind-set. If the development of hacking skills was connected with repeated practice and narrow interests [e.g., in line with the suggestion of either Ericsson and Faivre (1988) or Happé and Vital (2009) regarding the possible triggers of talent in autism], we would expect hackers to report poorer attention switching than non-hackers. Moreover, if poor mindreading was an indirect facilitator, we would expect hackers to report lower social, imagination, and communication skills than non-hackers. This part of the study mainly addresses question (a). Here, we are interested in establishing whether an association does exist or not between autistic traits and enrolment in an ethical hacking course in the general population; if such an association exists, it will be also possible to clarify whether some traits (e.g., detail-focus) may be more relevant than others. A sub-sample of individuals from both groups (hackers and non-hackers) was also tested with the systemizing quotient (*SQ*) questionnaire (Baron-Cohen et al., 2003; Goldenfeld et al., 2005; Wheelwright et al., 2006), aimed at classifying individuals for the strength with which they feel and express the drive to systemize. This further addresses question (a). Following Baron-Cohen et al.'s (2009) suggestion that the systemizing trait is crucial to the development of skills and interests in science and technology, we predicted significantly higher *SQ* scores in hackers than non-hackers.

Questions (b) and (c) were addressed by introducing behavioral tests in the design. The same subsample was also tested with two behavioral tasks: a prototypical hacking challenge centered around code-breaking skills and an additional security task focused on X-ray image interpretation skills. Code-breaking was chosen due to its being a prototypical hacking task, whereby individuals need to identify a strategy and/or a handle to break into a system (i.e., an unintelligible message obtained via a rule-based encryption algorithm), and reverse its encryption to obtain intelligible information. While other prototypical hacking tasks (e.g., website or computer systems penetration) would make sense only to participants with pre-existing programming knowledge, the logic of classical encryption can be easily explained to non-hackers. This made our code-breaking challenge suitable for both groups of participants. It required minimal knowledge of the encryption/decryption process and performance levels were predicted to vary depending on differences in familiarity with the task and drive/predisposition to understand and test the basic principles of cryptography. Differences in performance were thus expected to emerge at the group level depending on expertise (hackers vs. non-hackers) but, more importantly, within groups depending on individual systemizing scores. Indeed, although the hacking challenge did not require any complex mathematical calculations, it did require individuals to identify, understand, and apply transformation rules from plaintext to cypher-text and back to plaintext. As any exemplar code-breaking test (see also Lawson, 2005, for the use of a code breaking test in people with autism), our challenge involved the ability to recursively test hypotheses, and to detect pattern and regularities in apparently random strings of alphanumeric characters to identify structural or conceptual entry points enabling message decryption (e.g., Singh, 1999). Based on Baron-Cohen et al.'s (2009) systemizing model, we predicted that *SQ* would show a robust relation with hacking skills, whereas self-reported attention to detail—and possibly the *AQ* score—may be more loosely related to them (as detail focus or any other traits measured by the *AQ* may not suffice to develop systemizing skills). The security X-ray screening task was included as a specificity-control task because it bears no obvious relation to systemizing but it has been previously connected to self-reported attention to detail (Rusconi et al., 2015). We thus predicted a double dissociation between attention to detail and *SQ*, whereby the former is more related to performance in the X-ray image interpretation and the latter to performance in the hacking challenge; alternatively, attention to detail could be related to both the X-ray image interpretation task and the hacking challenge, whereas systemizing could be more specifically related to the hacking challenge.

## Methods

### Participants

One-hundred and fifty-nine volunteers (79 males and 80 females; 136 in the age range 18–24; 20 in the range: 25–34; 1 in the range: 35–44; 1 in the range 45–54, and 1 in the range 55–64) with no reported learning disabilities completed the *AQ*. Fifty-six of them (46 males and 10 females) were enrolled in an ethical hacking degree course (BSc or MSc level) or had recently completed it and henceforth will be referred to as *hackers*. The rest (103 respondents; 33 males and 70 females) were enrolled in or had recently completed a different type of degree course (mainly BSc or MSc psychology) and will be referred to as *non-hackers*. Fifty-nine volunteers from this initial sample responded to the subsequent recruitment call for the behavioral study. Data from 56 participants (34 females and 22 males; 51 in the age range 18–24 and 5 in the range 25–34) were eventually included in the analyses, due to incomplete data from three of the original participants. Of those 56 participants, 13 (10 males and 3 females) were hackers and 43 (12 males and 31 females) were non-hackers. The study was approved by the Ethics committee of Abertay University and all participants gave their informed consent online and in writing at the beginning of the testing sessions.

### Self-reports

#### Autism spectrum quotient (AQ)

An online version of the original *AQ* (Baron-Cohen et al., 2001) was administered via SurveyMonkey™. The *AQ* is an agile instrument for quantifying where individuals from the normal population are located on the continuum from autism to normality. It comprises 50 questions divided in five scales (10 questions for each scale) measuring social skills, attention switching, communication, imagination and attention to detail. Its reported test–retest reliability is *r* = 0.70 overall and its internal consistency levels are in the acceptable range within the social sciences (Field, 2009; Cronbach's alpha for *AQ* scales: Communication = 0.65; Social Skills = 0.77; Imagination = 0.65; Detail = 0.63; Attention Switching = 0.67; Baron-Cohen et al., 2001). In order to obtain a general *AQ* and disassociate heightened attention to detail from other autistic traits, all questions were used in the current study, even though our hypotheses focused on the attention to detail scale. Participants were asked to indicate their level of agreement by selecting one of four statements (“definitely agree,” “slightly agree,” “slightly disagree,” “definitely disagree”).

#### Systemizing quotient (SQ)

An online version of the original *SQ* was administered via SurveyMonkey™ comprising 60 questions (Baron-Cohen et al., 2003). Responses were collected on a four-point scale ranging from “strongly agree” to “strongly disagree,” similarly to the *AQ*. The *SQ* has good internal consistency, with a Cronbach's alpha coefficient of 0.79 (calculated with a sample including both neurotypical and autistic individuals; Baron-Cohen et al., 2003). Compared to the revised version of the questionnaire (Wheelwright et al., 2006), the original version is shorter but presents a stronger male bias, meaning that it may be more sensitive to the systemizing trait in males rather than females. We dealt with this issue by performing additional analyses to control for possible gender-related confounds.

### Behavioral tasks

#### Hacking challenge

The hacking challenge comprised a series of code-breaking tasks of increasing complexity. The first part of the challenge was developed in Cryptbench^©^ 1.0 (London South Bank University) as a guided tutorial, and included the presentation of six common encryption and decryption methods with instructions on how each method operates. Participants were challenged to decrypt six simple messages consisting of single words or a sentence and using codes of increasing complexity, one at a time. They were not allowed to move on to the next level of complexity before having correctly decrypted the easier message. The encryption/decryption methods were: ASCII, Binary, Hexadecimal, Addition to ASCII-value, Addition with Modulo shift, and Statistical Decoding.

**Figure 1:**
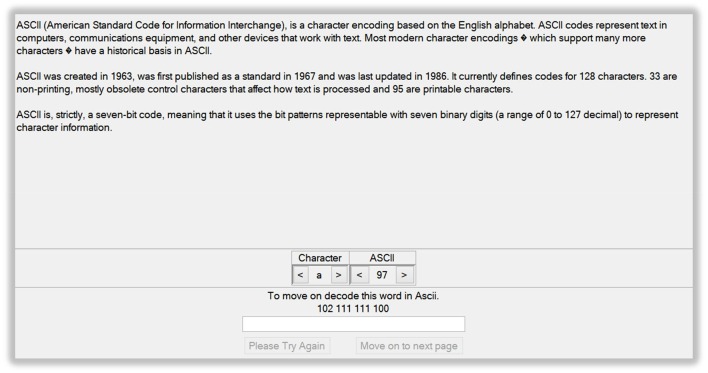
**Screenshot from the Cryptbench© interface for level 1 (ASCII coding)**.

The first level introduced ASCII coding, a type of character-to-number encoding based on the English alphabet. In the tutorial, a list of letters and a list of numbers in decimal notation were provided whereby each number represented a letter in ASCII code. By scrolling through the letter/number lists, participants were to find the letters making up the word provided by the programmer in ASCII code (Figure 1). After typing the correct word in a textbox, participants could move onto the second level. The second and third levels introduced binary and hexadecimal coding. In Mathematics and Computer Science, the binary or base-2 numeral system is a positional system that represents numeric values using only two different symbols: typically 0 and 1. For example, the decimal numeral 27 is 11011 in binary notation. The binary system is the internal operative language of almost all computers and computer-based devices. A binary digit is referred to as a bit and a consecutive series of eight digits is referred to as a byte. Hexadecimal, base-16, or simply hex, is a numeral system with a base of 16 usually written using the symbols 0–9 and A–F or a–f. For example, the decimal numeral 79 is 4F in hexadecimal. Hexadecimal is primarily used in computing to represent a byte, whose 256 possible values can be represented with only two digits in hexadecimal notation. In the Cryptbench level 2 and 3 tutorials, participants were encouraged to scroll along through the hex, ASCII and binary codes, and discover the letters that made up two words encoded in hex. The correct words had to be then typed into a textbox to move on to the next levels. Next, the concept of encoding by simple addition to ASCII-values and then back into a new text was introduced. To move on to the next level, participants had to transform an encoded word into ASCII, identify the number that was added to the original ASCII code, identify the original word and type it into a textbox. The following level showed how adding to the ASCII-value of a text and then adding a modulo shift to the resulting value, the decryption is made more difficult. In the final level, the concept of letter frequency analysis was introduced. This was aimed to help identify patterns in an encoded text message consisting of one sentence. The user was thus encouraged to replace encoded letters with potential candidates based on their frequency in text. This would eventually help decrypt the message.

The six guided decrypting tasks were followed by three more advanced tasks that had been appositely created for this study. Participants received three differently encrypted messages, along with information about the methods used to encrypt each of them, and a glossary (see Appendix A). The first hacking challenge involved a combination of Caesar cipher and reversed text. The Caesar cipher is one of the earliest encryption methods and involves replacing plain text letters with letters from the same alphabet but shifted by *n* places (in this case an offset of 2 was applied). The encoded message was also flipped in reverse, so the participant would then have to flip the message round to read in the correct order. The second challenge was more complex and used a combination of the Caesar cipher (offset of 7), ASCII coding (which replaces each letter with a decimal numeral according to the ASCII code) and binary coding (which translates decimal numerals into binary numerals). The third challenge was the most complex and used a combination of the Atbash cipher, the Caesar cipher (offset of 14), and the homophonic substitution cipher. The Atbash cipher is a very specific case of substitution cipher where the letters of the alphabet are reversed (i.e., all As are replaced with Zs, Bs are replaced with Ys, etc.). The homophonic substitution cipher involves replacing each letter with a variety of substitutes, the number of potential substitutes being proportional to the frequency of the letter in a given language. For example the letter “a” accounts for roughly 8% of all the letters in English, so eight different symbols may be used to represent it. Every time an “a” appears in the plaintext, it is randomly replaced with one of the eight symbols, so that by the end of the process each symbol accounts for roughly 1% of the cipher text. This serves the purpose to undermine the utility of frequency clues to decrypt the message.

#### Security X-ray screening task

We used the same stimuli and security X-ray screening task as described in Rusconi et al. (2015, pp. 3–6). In essence, the task consisted of responding as quickly and accurately as possible to whether or not images of X-rayed bags contained a threat (e.g., bullets, gas grenades, handguns, or knives). In the current study, images were presented and responses recorded with E-Prime professional 2.0 (Psychology Software Tools, Inc.) on laboratory PCs.

### Procedure

All participants who had completed the *AQ* online and provided their contact details were invited to take part in a laboratory testing session. Those respondents, who agreed, were then tested in groups in the ethical hacking laboratories at Abertay University. During testing, each of them had access to a PC with restricted connection to the web (i.e., only relevant websites could be accessed). Participants were not seated next to one another and they were constantly monitored to ensure that no exchange of information occurred during testing.

Including set up, instructions and debriefing, each laboratory session lasted about 1 h. The order of tasks (*SQ*; hacking challenge; X-ray screening task) was counterbalanced between participants.

During the hacking challenge, participants were instructed to first complete the Cryptbench levels, consisting of six alphanumerical code-breaking tasks. These were then followed by the three advanced code-breaking tasks, to be solved in the same order in which they were presented. Participants were allowed a maximum of 15 min to complete the Cryptbench series, after which the experimenter instructed them to move on to the advanced challenges, with a maximum of 10 min allowed for completion. These maximum completion times were decided on the basis of previous pilot testing. Each individual task was presented with a series of instructions allowing the participant to be aware of the expectations and the time constraints which came along with each category of tasks. Overall completion times and level achieved were recorded.

No time limits were set for completing the *SQ*, which was accessed online from the link provided by the experimenters. Most participants completed it in < 10 min. No time limits were set for completing the X-ray screening task either. This task was almost invariably completed within 10 min, as participants were encouraged not to take too long a break between the two experimental blocks. At least two experimenters were present and available to answer any questions and to monitor participants throughout the session.

### Data analysis

Statistical analyses were performed using IBM-SPSS v.22 and Bonferroni-Holm corrections for multiple comparisons were applied in exploratory analyses. The original binary scoring was used for the *AQ* data (Baron-Cohen et al., 2001), whereby for each item the autistic options (whether expressed “strongly” or “slightly”) received a score of 1, and the non-autistic options (whether expressed “strongly” or “slightly”) received a score of 0. Thus, the total *AQ* score theoretically ranged between 0 and 50. The original scoring method was also used for the *SQ* data (Baron-Cohen et al., 2003), whereby for each item the autistic options received a score of either 2 (if expressed “strongly”) or 1 (if expressed “slightly”) and the autistic options (whether expressed “strongly” or “slightly”) received a score of 0. Due to the presence of 20 filler items, which are excluded before the scoring procedure, the *SQ* score theoretically ranged between 0 and 80. Due to violations of the normality assumption (see Results Section) from all total and subscale scores, questionnaire data were analyzed with non-parametric statistics (Field, 2009). A series of Spearman's correlations was performed to test for relations between total *AQ, AQ* subscales, and *SQ* scores. To test the hypothesis of a relation between *AQ*, Attention to Detail scores, and interests in hacking, we performed Mann–Whitney U tests between groups (hackers vs. non-hackers) having the *AQ* and its subscale scores as dependent variables. Hacking performance was measured as the number of code-breaking challenges that had been solved within the available time (thus represented by an individual score theoretically ranging between 0 and 9) and overall solving time (theoretically ranging between 0 and 25 min). Each code-breaking challenge was deemed as solved if the participant had successfully decoded the corresponding message (e.g., for the challenge shown in Figure 1, participants were expected to write the word “food” on their response sheet). To assess whether *AQ*, Attention to Detail or *SQ* scores may relate to hacking performance, we calculated Spearman's correlations between questionnaire scores and hacking performance in the sample of 56 participants (hackers and non-hackers) who completed both the questionnaires and the hacking challenge. Additional analyses were performed on subsamples of participants to control for possible confounding effects of expertise and gender, and on performance in the X-ray screening task to assess whether the relation that we found between *SQ* and hacking performance could be explained away by general ability factors.

## Results

### Self-reports

#### AQ

Overall, the initial sample of 159 respondents obtained a median *AQ* score of 16 (Inter-Quartile Range, *IQR* = 8) and median scores of 2 (*IQR* = 2) in the Social Skills subscale, 4 (*IQR* = 3) in the Attention Switching subscale, 6 (*IQR* = 2) in the Attention to Detail subscale, 2 (*IQR* = 3) in the Communication subscale, and 2 (*IQR* = 2) in the Imagination subscale. All distributions showed significant deviations from normality according to Kolmogorov–Smirnov tests (all *p*s < 0.029). To assess whether the Attention to Detail trait was associated with other typical autistic traits in our sample of participants, we performed a series of Spearman's rank correlation tests between the *AQ* subscales (see Table 1). After applying the correction for multiple tests, no significant correlation was detected between the Attention to Detail scale and any of the other subscales.

**Table 1:** **Spearman's rank correlation coefficients and significance levels are reported for the total ***AQ*** score and for every subscale (***N*** = 159)**.

**Scale**	**Attention switching**	**Attention to detail**	**Communication**	**Imagination**
Social skills	0.33^*^	0.07	0.60^*^	0.23^*^
	0.000	0.390	0.000	0.003
Attention switching		−0.01	0.45^*^	0.11
		0.974	0.000	0.175
Attention to detail			0.13	0.16
			0.097	0.038
Communication				0.23^*^
				0.004

*Asterisks indicate two-tailed tests that remained significant after Bonferroni–Holm correction (min α = 0.005)*.

Mann–Whitney U tests for independent samples were then used to test differences between groups (hackers: *N* = 56; non-hackers: *N* = 103). These revealed that the two groups differed in their total *AQ* (hackers: median = 18.5, *IQR* = 8; non-hackers: median = 15, *IQR* = 8) and Attention to Detail scores (hackers: median = 7, *IQR* = 2.75; non-hackers: median = 6, *IQR* = 3) but not in the other subscales (see Table 2). Whereas non-hackers' scores were, on average, similar to the scores reported by Baron-Cohen et al. (2003) for the general population (we report parametric statistics for a direct comparison with Baron-Cohen et al.'s norms: non-hackers' mean *SQ* score = 25.58, 95% *CI* = 22.25–28.91; range = 7–57), hackers' scores were markedly above average (hackers' mean *SQ* score = 41.38, 95% *CI* = 34.46–48.31; range = 27–70). It remains to be seen whether this pattern of scores is similar to that obtained by individuals from other STEM disciplines or if it is characteristic of hackers. Based on their total *AQ* score (mean = 17.54, 95% *CI* = 12.96–22.15; range = 7–36), hackers seem closer to engineers than computer scientists/mathematicians, however their Attention to Detail score (mean = 6.69, 95% *CI* = 5.61–7.78; range = 3–9) outranks that of all the other STEM disciplines (Baron-Cohen et al., 2001).

**Table 2:** **Descriptive statistics and Mann–Whitney tests for independent samples (hackers vs. non-hackers) are reported for the total ***AQ*** score and for every subscale**. These statistics and tests are shown both for the initial group (total N = 159; hackers: N = 56, non-hackers: N = 103) and for the subgroup of participants who completed also the SQ questionnaire and lab tests (total N = 56; hackers: N = 13, non-hackers: N = 43).

**Questionnaire/Subscale**	**Median (*IQR*)**	**Median (*IQR*)**	**Median (*IQR*)**	**U (*SE*)**	**Sig (2-tailed)**.
	**All**	**Hackers**	**Non-hackers**		
***AQ***					
*N* = 159	16 (8)	18.5 (8)	15 (8)	3714 (277)	0.003^*^
*N* = 56	16 (6)	16 (6.5)	16 (6)	301 (51)	0.676
**SOCIAL SKILLS**					
*N* = 159	2 (2)	2 (2)	2 (1)	3346 (267)	0.083
*N* = 56	1 (2)	1 (2)	1 (2)	316 (50)	0.461
**ATTENTION SWITCHING**					
*N* = 159	4 (3)	4 (2)	4 (3)	3022 (273)	0.613
*N* = 56	4 (3)	3 (1.5)	4 (3)	186 (50)	0.061
**ATTENTION TO DETAIL**					
*N* = 159	6 (2)	7 (2.75)	6 (3)	3880 (274)	0.000^*^
*N* = 56	6 (2.8)	7 (2.5)	6 (3)	354 (51)	0.144
**COMMUNICATION**					
*N* = 159	2 (3)	2 (3)	2 (2)	3374 (273)	0.073
*N* = 56	2 (2.8)	1 (5)	2 (2)	254 (50)	0.621
**IMAGINATION**					
*N* = 159	2 (2)	2 (3)	2 (2)	3284 (272)	0.140
*N* = 56	2 (2)	2 (3.5)	2 (2)	257 (50)	0.663
***SQ***					
*N* = 159	–	–	–	–	–
*N* = 56	27.5 (16.8)	38 (12)	24 (15)	481 (51)	0.000^*^

*Asterisks indicate two-tailed tests that remained significant after Bonferroni–Holm correction (min α = 0.008). IQR, Inter-Quartile Range; U, test statistic; SE, standard error; Sig., significance*.

#### SQ

The following analyses were performed on the restricted sample of participants (total *N* = 56; hackers: *N* = 13; non-hackers: *N* = 43) for whom *SQ* scores were available. Along the systemizing dimension, our sample obtained an overall median score of 27.5 (*IQR* = 17). A series of Spearman's correlations were performed to tests for possible relations between the *AQ* total score or its component subscale scores and the *SQ* score (see Table 3). The *SQ* score was positively correlated with Attention to Detail (ρ = 0.39, *p* = 0.003) and negatively correlated with Attention Switching (ρ = –0.36, *p* = 0.007). These correlations remained significant even after applying a Bonferroni–Holm correction. No other significant correlations were found. Non-hackers obtained a median *SQ* score of 24 (*IQR* = 15) and hackers obtained a median *SQ* score of 38 (*IQR* = 12). A Mann–Whitney U test confirmed that hackers' *SQ* median score was significantly higher than non-hackers' (*U* = 481, *SE* = 51, *p* < 0.001; see Table 2).

**Table 3:** **Spearman's rank correlation coefficients between the ***SQ*** and the ***AQ*** scores (total and subscales) and their significance levels are reported (***N*** = 56)**.

**Score**	***AQ* total**	**Social skills**	**Attention switching**	**Attention to detail**	**Communication**	**Imagination**
*SQ*	0.24	0.21	−0.36^*^	0.39^*^	0.25	0.23
	0.079	0.122	0.007	0.003	0.062	0.083

*Asterisks indicate significant two-tailed tests after Bonferroni–Holm correction (min α = 0.008)*.

### Behavioral tasks and self-reports

**Figure 2:**
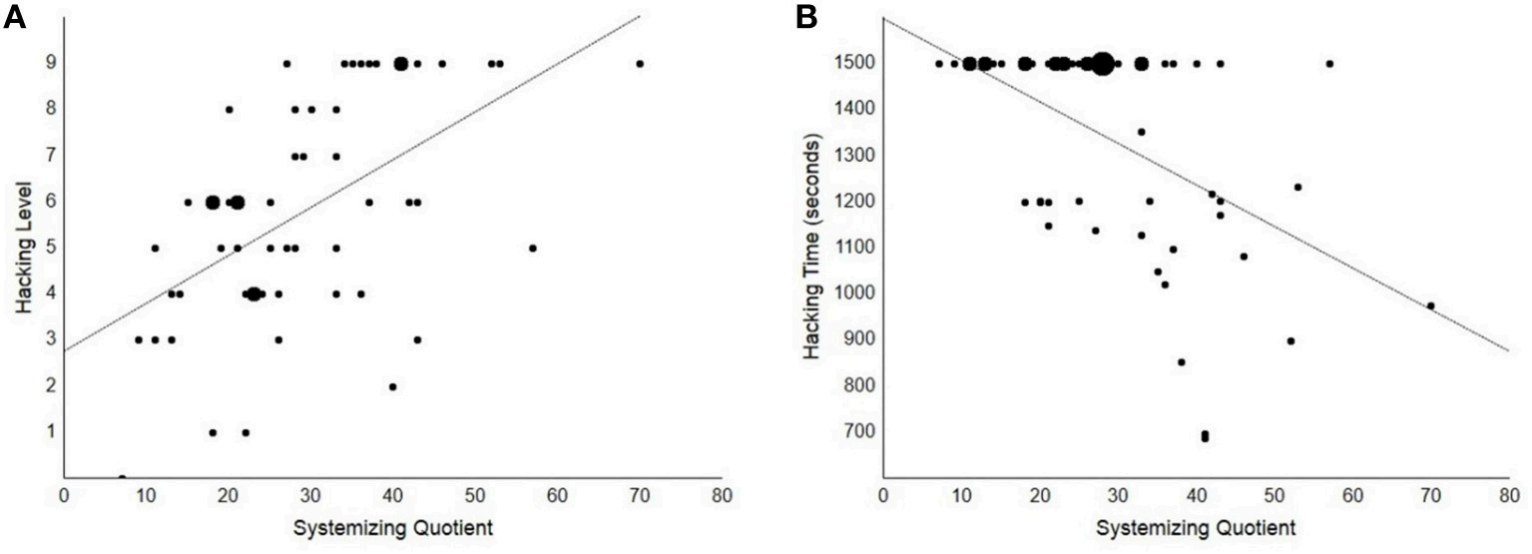
**Scatterplots showing the significant correlations between SQ and hacking performance (ps < 0.001). (A)** Scatterplot showing a significant positive correlation (ρ = 0.55, *p* < 0.001) between *SQ* and hacking level. This correlation cannot be accounted for by expertise or gender bias alone (see main text). Dot size is directly proportional to the number of cases placed at the same coordinates. **(B)** Scatterplot showing a significant negative correlation (ρ = −0.47, *p* < 0.001) between *SQ* and hacking time. This correlation appeared less robust than the correlation between *SQ* and with hacking level as it was not significant when controlling for the effects of expertise and gender bias (see main text). This may be also due to the majority of participants having used up all the available time to solve the hacking challenge, with considerably reduced variability in the hacking time data compared to the hacking level data.

**Figure 3:**
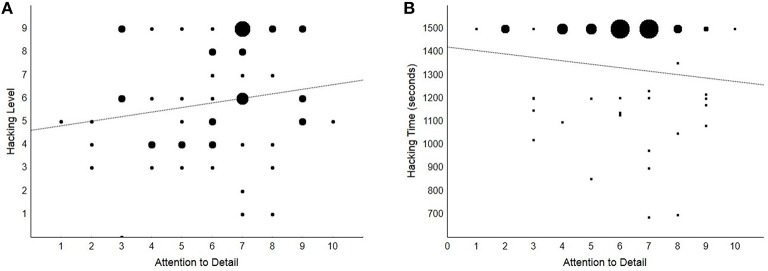
**Scatterplots showing the non-significant correlations between Attention to Detail and hacking performance (***p***s > 0.17)**. Dot size is directly proportional to the number of cases placed at the same coordinates. **(A)** Hacking level is shown as a function of Attention to Detail. **(B)** Hacking time is shown as a function of Attention to Detail.

Overall, participants obtained a median score of 6 (*IQR* = 4) at the hacking challenges. Hackers reached a significantly higher level (median = 9, *IQR* = 0.50) than non-hackers (median = 5, *IQR* = 2; Mann–Whitney *U* = 504, *SE* = 51, *p* < 0.001), however no significant correlation was found between Attention to Detail and hacking performance (Spearman's ρ = 0.19, *p* = 0.167; see **Figure 3A**) or between *AQ* and hacking performance (ρ = 0.05, *p* = 0.686). On the other hand, a significant moderate correlation was found between *SQ* and hacking performance (ρ = 0.55, *p* < 0.001; see Figure 2A). Overall, our participants were engaged in the hacking challenges for all the time available (median = 25 min, *IQR* = 5; no difference between groups). We did not find a significant relation between Attention to Detail or *AQ* and hacking time (both *p*s > 0.311; see Figure 3B). The correlation between *SQ* and hacking time was significant and of negative sign (ρ = −0.47, *p* < 0.001; see Figure 2B), which indicates that the relation between *SQ* score and hacking level is not due to speed-accuracy trade-off.

Because *SQ* scores co-vary with expertise, we checked if the correlations remained significant after exclusion of the 13 ethical hackers from the sample. Despite the decrease in sample size, a significant positive correlation was still found between the *SQ* score and hacking performance (ρ = 0.34, *p* = 0.028), whereas the negative correlation between *SQ* and hacking time did not reach significance (ρ = −0.21, *p* = 0.169). In other words, the relation between *SQ* and hacking level was present and significant even after removing the effect of expertise. A similar pattern was also found when considering the restricted sample of hackers only, with a significant positive correlation between *SQ* score and hacking performance (ρ = 0.61, *p* = 0.026) and a non-significant negative correlation between *SQ* and hacking time (ρ = −0.45, *p* = 0.122). This further confirms that differential expertise is not sufficient to account for the reported relation between *SQ* and hacking performance.

The original version of the *SQ* is known to be especially affected by a male bias (Baron-Cohen et al., 2003; Wheelwright et al., 2006), and a significant difference between genders could be detected in our sample, with males obtaining significantly higher scores than females (Mann–Whitney *U* = 652, *SE* = 60, *p* < 0.001; males: median *SQ* = 36, *IQR* = 10; females: median *SQ* = 21.5, *IQR* = 11). To check whether the male bias could act as a confound, we probed again the relation between *SQ* score and hacking performance after exclusion of the 22 males in our sample. The relation between *SQ* and performance was still significant and in the expected direction for hacking level; it was close to significance for hacking time (*N* = 34: hacking performance ρ = 0.48, *p* = 0.005, hacking time ρ = −0.33, *p* = 0.059). The relation between *SQ* and hacking was still fully significant (though for hacking times rather than performance level) when considering the sample of males only (*N* = 22: hacking performance ρ = 0.28, *p* = 0.201, hacking time ρ = −0.43, *p* = 0.046).

Lastly, we assessed the specificity of the relation between *SQ* and hacking performance by testing whether high *SQ* scores were also associated with better performance in a security X-ray screening task. Overall, participants responded correctly to 76% (*SE* = 0.98) of the trials and with an average latency of 1368 ms (*SE* = 63). No significant correlations were found between *SQ* score and accuracy or reaction times in the X-ray screening task (X-ray screening overall accuracy, accuracy in threat-present and accuracy in threat-absent trials: all *p*s > 0.163; overall RTs, RTs in threat-present trials and in threat-absent trials: all *p*s > 0.209), which are the same indices for which we detected significant correlations between *SQ* score and hacking performance instead. In contrast, and notwithstanding the smaller sample size compared to Rusconi et al. (2015) and our use of non-parametric statistics, at least a significant correlation was found between the Attention to Detail score and an index of accuracy in the X-ray screening task (threat absent trials: ρ = 0.30, *p* = 0.026; all other *p*s > 0.070).

## Discussion

In this study, we recruited two groups of individuals, hackers and non-hackers, without learning disabilities. We measured the autistic traits of interest via two self-report questionnaires suitable for adults of normal intelligence: the *AQ* and the *SQ* (Baron-Cohen et al., 2001, 2003). We measured hacking skills via a tailored hacking challenge that did not require previous expertise but would likely benefit from familiarity and individual predisposition to develop a hacker's mind-set. We also included a control task involving security X-ray image interpretation, which has no obvious relation with the hacker mind-set but had been previously related to piecemeal attention as measured within the *AQ* (e.g., Rusconi et al., 2015). Its presence enables us to test the specificity of trait measures known to belong to the same core family of traits (Baron-Cohen et al., 2009) in predicting performance within the same participants.

By taking into account the available evidence on the hacker mind-set and the theoretical proposals relating autistic traits and talent development, we articulated the following predictions: (a) hackers' *AQ*, Attention to Detail and *SQ* scores will be significantly higher than non-hackers'; Attention Switching and scores in other *AQ* subscales implying mentalizing skills might also be significantly higher in hackers than in non-hackers; (b) Attention to Detail and *SQ* scores will be significantly related with hacking skills; (c) whereas *SQ* scores will be specifically related with hacking skills (i.e., they will not be also related with the X-ray screening task), Attention to Detail may play a more general role and be related with both hacking and X-ray image interpretation skills.

The data showed that (a) hackers reported significantly higher levels for *AQ*, Attention to Detail and *SQ* than non-hackers, whereas they reported the same levels as non-hackers for Attention Switching and other skills involving mentalizing; (b) *SQ* scores but not Attention to Detail scores showed a robust relation with performance in the hacking challenge, which cannot be explained by expertise or gender bias only; (c) *SQ* scores were not related with performance in the control X-ray screening task, which was instead related with Attention to Detail scores. Our predictions were thus partly confirmed.

We conclude that there may be much more behind the idea of a relation between autistic traits and hacking than anecdotes and popular media portraits. As far as the relation of *AQ* scores and Attention to Detail with hacking is concerned, we replicated and extended with a sample of ethical hacking students vs. controls (total *N* = 159) Schell and Melnychuk's (2011) findings, which were based on self-reports of hacking conference attendees. We compared hackers' *AQ* scores to a matched control group of non-hackers and showed that the source of the significant difference was mainly due to differences in the trait measured by Attention to Detail. Additionally, we found that hackers reported significantly higher systemizing than non-hackers. Non-hackers' scores were on average similar to the scores reported by Baron-Cohen et al. (2003) for the general population, whereas hackers' scores were markedly above average. It remains to be seen whether this pattern of scores is similar to that obtained by individuals from other STEM disciplines or if it is characteristic of hackers. Based on their total *AQ* score, hackers seem closer to engineers than computer scientists/mathematicians, however their Attention to Detail score outranks that of all the other STEM disciplines (Baron-Cohen et al., 2001). This could result from a systematic self-report bias (i.e., hackers prefer to portray themselves by emphasizing the positive and downplaying the negative autistic traits) or from a genuine trade-off between the positive and the negative autistic traits in the hacker population. It is possible that hackers are also characterized by good mentalizing skills. Indeed, social engineering features prominently in the hacker toolset (see e.g., Mitnick, 2002). Note that the differences mentioned above have emerged with our larger groups. We did not find any significant difference in Attention to Detail or *AQ* in our smaller group of hackers and non-hackers who volunteered to participate in the laboratory session. Lack of a significant difference may be due to insufficient power (total *N* = 56) in the case of the Attention to Detail score, as the median scores of the two subgroups are identical to the median scores of their corresponding larger groups. Instead it may be due to self-selection bias related to volunteering for group testing in the laboratory in the case of the *AQ* score. Indeed, the median *AQ* scores of hackers and non-hackers were identical, when looking at the subgroups. The characteristics of our laboratory testing session may have selectively discouraged those with higher autistic traits overall.

In our study we also assessed the correlation between self-reported autistic traits and behavioral performance in two different tasks: a hacking challenge and a security X-ray screening task. The hacking challenge was set up in such a way that both hackers and non-hackers could easily engage with it and received all the necessary information to progress through levels of increasing difficulty. Here, we found performance differences between groups and, within each group, between individuals. The difference between groups may reflect a combined effect of expertise, familiarity with the task and systemizing. The difference between individuals and within each group may be a genuine reflection of inter-individual differences in systemizing. Notably, systemizing was operationalized as a trait. That is, we have asked participants to report how well typical systemizing behaviors, thinking habits, and choices match to their own habitual choices, thoughts, and behaviors. This naturally increases the likelihood that higher systemizers, even in the group of non-hackers, may be more familiar than lower systemizers with the rationale of the code-breaking task (e.g., because they enjoy solving puzzles in their free time). It is unlikely, however, that they had been exposed to the same tasks that were included in our hacking challenge (a) because the most difficult ones were created appositely for this study; (b) the Cryptbench© app has been developed for introducing basic cryptography principles to computer science students.

To control for the specificity of the relation between systemizing and code-breaking, we also tested our participants with an X-ray screening task. It was argued and shown elsewhere that X-ray image interpretation, due to the peculiar challenges it poses to the visual system, may be positively correlated with field-independence in the visual domain (Rusconi et al., 2012, 2015). In our testing protocol, we did not provide extensive training with a large library of security threats (a process in which the systemizing trait might have played a role), but we did test participant' ability to quickly interpret a series of novel and cluttered images on the spot. Here, systemizing did not predict performance, suggesting specificity in its relation with the hacking task. On the other hand, we found some evidence of a relation between piecemeal attention and the X-ray screening task which indicates a possible double dissociation. This resembles a weak double dissociation as the small positive correlation between Attention to Detail and hacking level may be statistically significant with a larger sample size. This would appear consistent with the fact that our design did not detect differences between the hackers vs. non-hackers subgroups in the Attention to Detail trait but not in the systemizing trait (see above). Based on the available evidence, we thus maintain that systemizing is not related to X-ray image interpretation but can be a moderate predictor of code-breaking skills. We also expect piecemeal attention to be not as good a predictor of hacking skills as systemizing, were it to be found related with hacking performance in larger samples of participants. In line with Baron-Cohen et al.'s (2009) model of the relation between autistic traits and talent in systemizing domains, hyper-attention to detail may be related to code-breaking by virtue of its relation with systemizing. Finally, the positive correlation between *SQ* and Attention to Detail scores appears consistent with models emphasizing the role of detail focus in the relation between autistic traits and talent (Baron-Cohen et al., 2009; Happé and Vital, 2009; Vital et al., 2009). But the anti-correlation between *SQ* and Attention Switching is inconsistent with models emphasizing the role of narrow interests and repeated practice (Ericsson and Lehmann, 1996) in the development of systemizing skills.

Overall, our study offers an original take on autistic traits and their potential in neurotypicals. Due to the relatively small sample sizes available, to its limited range of tasks and the different gender ratios in our two groups (hackers vs. non-hackers), it offers seed evidence to guide future research. In future research, for example, it would be useful to probe whether the relation that we established between systemizing and code-breaking may generalize to a wider range of hacking tasks. If a larger sample of participants was available, it may be possible to better describe the relation between piecemeal attention and systemizing and to establish their relative weight in predicting performance across security tasks. The ethical hacking group who volunteered to complete the *SQ* and behavioral tasks in the laboratory was particularly small; furthermore it may not have been representative of the population of ethical hackers, due to volunteering bias. However, the focus of this part of the study was on individual differences and their relation with performance rather than on differences between groups. The part investigating differences between groups could count on larger sample sizes for both hackers and non-hackers, who responded online. Noteworthy, this method of relatively unconstrained sampling led to a sex ratio in the hackers group (3–4 males : 1 female) that was similar to the sex ratio of autism, and to a reversed sex ratio in the non-hackers group. In future studies, the use of balanced groups could help explore sex differences in skills and predispositions. Finally, in this study we measured traits via self-reports. Questionnaires are a very convenient and agile way to identify where an individual stands along a trait continuum; even if research participants had no reason to respond strategically, their identification with a specific group (e.g., ethical hackers) may have triggered stereotyped responses based on shared group values rather than responses based on genuine individual characteristics. This would lead to artificial differences between groups in self-reported systemizing or attention to detail. On the other hand, if participants' responses to the questionnaires did not reflect genuine individual differences, a null correlation would be expected between self-reported systemizing and hacking skills within each group.

From a translational viewpoint, our study suggests that certain autistic traits may correlate with better performance in information security jobs. It also points to the possible utility of the *SQ* for personnel selection and provides partial validation for current trends promoted by social enterprises. Social entrepreneurs have been focusing on autistic traits in people with autism rather than in the neurotypical population in recent years. A few successful start-ups have emerged, whose primary aim is to introduce autistic people into the world of work (see e.g., the German-based *auticon*)—most often, into information technology and financial jobs (Putzier, 2013). It has been recognized that people with autism may be equipped with unique resources (provided that their transition into employment is eased by assistance to accommodate atypical interpersonal styles and sensory processing). By focusing on autistic traits in people without learning disabilities, we extend the reasoning to the wider population and offer evidence that could ease the process of finding a good match between individual characteristics and the job market on a larger scale. We have also shown that systemizing and/or attention to detail are not obviously related with the negative traits of autism in the general population. Whereas, our findings may lead to relatively straightforward applications with neurotypicals, unfortunately the same cannot be said of people with autism due to the concomitant presence of negative traits. This points to the need of similar and additional research in people with autism.

## Author contributions

IH, conducted the study; SB, co-wrote the manuscript; DM, conducted the study and provided testing materials; CM, provided testing materials and lab space; ER, supervised the study.

### Conflict of interest statement

The authors declare that the research was conducted in the absence of any commercial or financial relationships that could be construed as a potential conflict of interest.

## Appendix A

### Subject document (page 1): encrypted messages

#### Test 1—easy

Methods used—Caesar cipher, Reversed Text

Cipher to be decoded “TGJRKE GJV FGXNQU WQA UPQKVCNWVCTIPQE”

#### Test 2—medium

Methods used—Caesar cipher, decimal coding, binary coding

Cipher to be decoded

“01001100010001010100101001001100010100110101001101001100010101010100000101011010

010000010100001001001101010011010100100101000010010000010101010101010110010001000101001101001100010000010101101001000001010000100101100101010101010000100101011101000001010011110100110001001111010011000100100001000001”

#### Test 3—hard

Methods used—Caesar cipher, Atbash cipher, homophonic substitution cipher

Cipher Text to be decoded—“33 22 12 02 88 58 06 60 07 35 04 21 72 61 65 67 34 57 62 77 47 62 04 74 79 43 23 24 26 09 19 20 62 73 38 65 55 42”

### Subject document (pages 2 and 3)—additional information

#### Caesar cipher

According to Suetonius, Caesar simply replaced each letter in a message with the letter that is three places further down the alphabet. Cryptographers often think in terms of the plaintext alphabet as being the alphabet used to write the original message, and the cipher text alphabet as being the letters that are substituted in place of the plain letters. When the plaintext alphabet is placed above the cipher text alphabet, as shown below, it is clear to see that the cipher text alphabet has been shifted by three places. Hence this form of substitution is often called the Caesar Shift Cipher. A cipher is the name given to any form of cryptographic substitution, in which each letter is replaced by another letter or symbol. The alphabet can be shifted up to 25 places, but shifting a letter 26 places takes it back to its original position, and shifting it 27 places is the same as shifting it 1 place. So there are 25 keys.

#### Reversed text

By reversing text characters are shifted back to front making the first part of the message come last and the last part come first. A useful technique is to start from the end and work your way back.

#### Binary coding

Binary is one of the simplest of all number systems because it has only two numerals: 0 and 1. In the decimal system (with which most people are accustomed), you use 10 numerals: 0 through 9. In binary, you have only two numerals rather than ten, which is why binary numbers look somewhat monotonous, as in 110011, 101111, and 100001. For each letter in the alphabet there are eight binary digits, this fun fact will help when it comes to segregating the code. The Table A1 below will help with your decoding.

**Table A1:** **ASCII alphabet characters**.

**Symbol**	**Decimal**	**Binary**	**Symbol**	**Decimal**	**Binary**
A	65	01000001	a	97	01100001
B	66	01000010	b	98	01100010
C	67	01000011	c	99	01100011
D	68	01000100	d	100	01100100
E	69	01000101	e	101	01100101
F	70	01000110	f	102	01100110
G	71	01000111	g	103	01100111
H	72	01001000	h	104	01101000
I	73	01001001	i	105	01101001
J	74	01001010	j	106	01101010
K	75	01001011	k	107	01101011
L	76	01001100	l	108	01101100
M	77	01001101	m	109	01101101
N	78	01001110	n	110	01101110
O	79	01001111	o	111	01101111
P	80	01010000	p	112	01110000
Q	81	01010001	q	113	01110001
R	82	01010010	r	114	01110010
S	83	01010011	s	115	01110011
T	84	01010100	t	116	01110100
U	85	01010101	u	117	01110101
V	86	01010110	v	118	01110110
W	87	01010111	w	119	01110111
X	88	01011000	x	120	01111000
Y	89	01011001	y	121	01111001
Z	90	01011010	z	122	01111010

#### Atbash cipher

The Atbash cipher is a very specific case of a substitution cipher where the letters of the alphabet are reversed. In other words, all As are replaced with Zs, all Bs are replaced with Ys, and so on. Because reversing the alphabet twice will get you the actual alphabet, you can encipher and decipher a message using the exact same algorithm.

Example Plaintext: This is a secret message Ciphertext: Gsrh rh z hvxivg nvhhztv.

#### Homophonic substitution

The Homophonic Substitution Cipher involves replacing each letter with a variety of substitutes, the number of potential substitutes being proportional to the frequency of the letter. For example, the letter “a” accounts for roughly 8% of all letters in English, so we assign eight symbols to represent it. Each time an “a” appears in the plaintext it is replaced by one of the eight symbols chosen at random, and so by the end of the encipherment each symbol constitutes roughly 1% of the ciphertext. The letter “b” accounts for 2% of all letters and so we assign 2 symbols to represent it. Each time “b” appears in the plaintext either of the two symbols can be chosen, so each symbol will also constitute roughly 1% of the ciphertext. This process continues throughout the alphabet, until we get to “z,” which is so rare that is has only one substitute. In the example provided, the substitutes happen to be 2-digit numbers, there are between 1 and 12 substitutes for each letter, depending on the letter's relative abundance.

The point of offering several substitution options for popular letters is to balance out the frequencies of symbols in the ciphertext. Every symbol will constitute roughly 1% of the ciphertext. If none of the symbols appears more frequently than any other, then this cipher would appear to defy any potential attack via straightforward frequency analysis.

### Master document with keys

#### Test 1—easy

Methods used—Caesar cipher, reversed text

Phrase that is scrambled: “congratulations you solved the cipher”

Ceasar cipher (offset of two)—“EQPITCVWNCVKQPU AQW UQNXGF VJG EKRJGT”

Text has been flipped in reverse “TGJRKE GJV FGXNQU WQA UPQKVCNWVCTIPQE”

#### Test 2—medium

Methods used—Caesar cipher, decimal coding, binary coding

Phrase that is scrambled: “Excellent stuff but now let's turn up the heat”

Ceasar cipher (offset of seven)—“LEJLSSLUAZABMMIBAUVDSLAZ ABYUBWAOLOLHA”

Converted to decimal—“76 69 74 76 83 83 76 85 65 90 65 66 77 77 73 66 65 85 86 68 83 76 65 90 65 66 89 85 66 87 65 79 76 79 76 72 65”

Converted to binary—

“01001100010001010100101001001100010100110101001101001100010101010100000101011010010000010100001001001101010011010100100101000010010000010101010101010110010001000101001101001100010000010101101001000001010000100101100101010101010000100101011101000001010011110100110001001111010011000100100001000001”

#### Test 3—hard

Methods used—Caesar cipher, atbash cipher, homophonic substitution cipher

Phrase that is scrambled: “Amazing work you have cracked the last cipher”

Converted through Atbash cipher—“Znzarmt dlip blf szev xizxpvw gsv ozhg xrksvi”

Ceasar cipher (offset of 14)—“NBNOFAH RZWD PZT GNSJ LWNLDJK UGJ CNVU LFYGJW”

Cipher Text to be decoded—“33 22 12 02 88 58 06 60 07 35 04 21 72 61 65 67 34 57 62 77 47 62 04 74 79 43 23 24 26 09 19 20 62 73 38 65 55 42”
